# *Novosphingobium aromaticivorans* LigR coordinates transcription of genes involved in metabolism of multiple types of aromatics

**DOI:** 10.1128/msystems.01469-25

**Published:** 2025-11-28

**Authors:** Laura Rodríguez-Castro, Kevin S. Myers, Alexandra M. Linz, Erin L. Mettert, Walter Camp, Patricia J. Kiley, Daniel R. Noguera, Timothy J. Donohue

**Affiliations:** 1 Great Lakes Bioenergy Research Center, University of Wisconsin-Madison, Madison, Wisconsin, USA; 2 Wisconsin Energy Institute, University of Wisconsin-Madison, Madison, Wisconsin, USA; 3 Department of Biomolecular Chemistry, University of Wisconsin-Madison, Madison, Wisconsin, USA; 4 Department of Civil and Environmental Engineering, University of Wisconsin-Madison, Madison, Wisconsin, USA; 5 Department of Bacteriology, University of Wisconsin-Madison, Madison, Wisconsin, USA; Agroscope Standort Reckenholz, Zurich, Switzerland

**Keywords:** *Novosphingobium aromaticivorans*, lignin, aromatic metabolism, transcription factor, RNA-seq, DAP-seq

## Abstract

Aromatic compounds are a ubiquitous and diverse family of chemicals with functions as biomolecules, natural products, industrial chemicals, and pollutants. *Novosphingobium aromaticivorans* DSM 12444 uses multiple inducible pathways to catabolize H-, G-, and S-type aromatics that contain zero, one, or two methoxy groups, respectively. Here, we obtain a systems-level view of the transcriptional control of its aromatic metabolic pathways. Several *in vitro* analyses found that a *N. aromaticivorans* homolog of the *Sphingobium lignivoran*s SYK-6 transcription factor LigR bound genomic DNA upstream of genes involved in metabolism of multiple aromatic types. We found that a ΔLigR mutant had growth defects on all three types of aromatics as sole carbon sources. Transcriptomic analysis revealed that LigR was required to increase expression of gene products that function in metabolism of all three aromatic types. We also found that, in media containing both glucose and an aromatic carbon source, the ΔLigR mutant directed intermediates through alternative aromatic metabolic pathways. Protein-DNA binding assays showed that *N. aromaticivorans* LigR binds immediately upstream of promoters of genes involved in aromatic metabolism. We found that *N. aromaticivorans* LigR coordinates the expression of enzymes that function in the catabolism of H-, G-, and S-type aromatics, and that there are differences in the role of LigR in *N. aromaticivorans* and *S. lignivorans*. A comparative genomic analysis predicted that LigR homologs and the aromatic-metabolizing genes that it directly regulates are often co-localized in the genomes of Sphingomonadales, but often not found in this arrangement in many other known aromatic metabolizing bacteria.

**IMPORTANCE:**

The abundance and societal importance of aromatics have led to interest in developing biological catalysts that can use them as a renewable source of industrial chemicals. While the synthesis of proteins needed for aromatic metabolism is often regulated, we lack a systems-level understanding of how cells coordinate the use of these pathways. Here, we used DNA affinity purification sequencing, RNA-seq, and targeted metabolite analysis of the bacterium *Novosphingobium aromaticivorans* to understand the transcriptional regulation of enzymes needed to metabolize different aromatic types. Analysis of a *N. aromaticivorans* DNA-binding protein, LigR, illustrated how synthesis of enzymes that function in multiple aromatic pathways is controlled. We propose that the insight obtained from this systems-level view of aromatic metabolism could help engineer bacteria to produce industrial chemicals or remove toxic aromatics from the environment.

## INTRODUCTION

Conversion of abundant aromatic compounds into fuels or industrial chemicals is an important societal goal. In particular, the plant biopolymer lignin is a desirable source of aromatics (1) because ~50–70 million tons of it are generated each year (2), making it the largest renewable source of aromatic compounds on Earth. The lignin polymer is composed of three major aromatic subunits containing zero, one, or two methoxy groups attached to the aromatic ring (H-, G-, and S-type aromatics, respectively) (2, 3). This, plus the different inter-aromatic bonds of this heteropolymer, has made it difficult to generate individual chemicals from lignin (4). However, the identification of microbes that natively convert renewable aromatics into useful products provides an opportunity to develop approaches to achieve this goal. To accomplish this, we need a comprehensive, systems-level understanding of aromatic metabolism in microbes that could potentially be used for this purpose (4, 5).

*Novosphingobium aromaticivorans* DSM 12444, an Alphaproteobacterium isolated from a polyaromatic hydrocarbon-contaminated site (6, 7), has emerged as an important bacterium for studying aromatic metabolism. *N. aromaticivorans* cleaves several major lignin inter-subunit linkages and metabolizes multiple types of aromatics using enzymes that function in one or more pathways (8–11). Strains have been engineered to funnel H- and G-type aromatics *via* protocatechuic acid (PCA) into 2-pyrone-4,6-dicarboxylic acid (PDC), convert PCA into *cis,cis*-muconic acid *via* the catechol intradiol pathway, generate PDC from intermediates in S-type aromatic metabolism (5, 12), and accumulate carotenoids (13). Synthesis of *N. aromaticivorans* enzymes that function in these aromatic metabolic pathways is regulated (9, 14), and studies have identified transcription start sites (TSS) by mapping RNA species that contain triphosphates at their 5′ ends (14). However, systems-level information is needed to define this transcriptional network and use it to design improved production strains.

Previous analysis of *Sphingobium lignivoran*s SYK-6 showed that a LysR-family transcription factor, LigR, binds to promoter regions upstream of genes involved in the 4,5-extradiol branch of PCA catabolism *in vitro* (15). However, an *in vivo* role for *S. lignivoran*s SYK-6 LigR in aromatic metabolism was not observed since a mutant lacking this protein exhibits only a slight growth defect when using either vanillic acid (VA), an aromatic that generates PCA as an intermediate, or syringic acid (SA) as a sole carbon source (16).

Here, we characterized *N. aromaticivorans* LigR and found that it has a role in metabolism of multiple aromatic types. Using **D**NA **A**ffinity **P**urification-**seq,** DAP-seq (17), we identified two binding sites for the *N. aromaticivorans* LigR homolog in the genome (18). Additional *in vitro* studies allowed precise mapping of these binding sites to promoters for two flanking operons encoding proteins involved in *N. aromaticivorans* metabolism of H-, G-, and S-type aromatics. RNA-seq analysis of a ΔLigR mutant revealed it is an activator of aromatic-inducible genes. The effect of deleting *ligR* was assayed *via* growth experiments with H-, G-, or S-type aromatics and by monitoring extracellular concentrations of a targeted set of compounds in ΔLigR and the parent strain. Our data lead us to predict that the route used to direct aromatic pathway intermediates through PCA and 3-O-methylgallic acid (3-MGA) depends on *N. aromaticivorans* LigR. Analysis of bacterial genomes for LigR homologs and genes that are regulated by this protein in *N. aromaticivorans* reveals a conserved co-localization of genes that flank *ligR,* primarily in Sphingomonadales. Altogether, our systems-level analysis of *N. aromaticivorans* LigR function provides unexpected strategies to reroute aromatic metabolites into products.

## RESULTS

### *N. aromaticivorans* LigR (SARO_RS14285) binds genomic DNA *in vitro*

**Fig 1:**
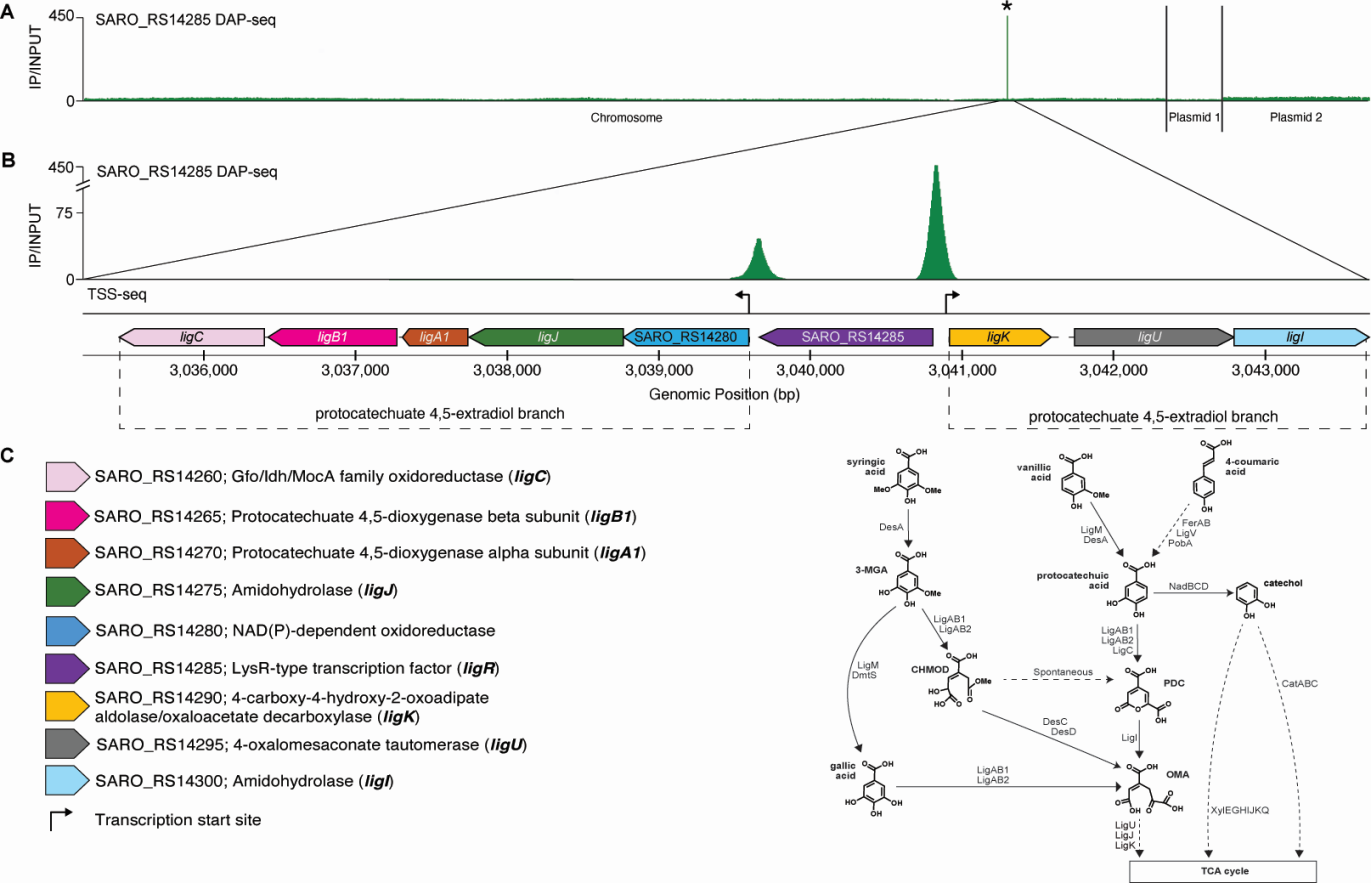
*In vitro* binding of a purified recombinant LigR protein to the *N. aromaticivorans* chromosome and its two plasmids. Panel **A**: The green trace (IP/INPUT) reports the ratio of normalized read counts in the LigR immunoprecipitated (IP) sample compared to those in a control sample lacking this protein (INPUT). Higher IP/INPUT values indicate increased frequency of a recombinant LigR protein binding to genomic DNA *in vitro*. The chromosome and the plasmids (pNL1 and pNL2) of strain DSM 12444 are shown (accession numbers: NC_007794.1 [chromosome], NC_009426.1 [pNL1], and NC_009427.1 [pNL2]). Panel **B**: Genes in the vicinity of two statistically significant *in vitro* DNA-binding sites for LigR (indicated by the asterisk (*) in panel A). The arrows represent previously mapped aromatic-inducible transcription start sites (14). Panel **C**: Annotated or known functions in aromatic metabolism of gene products flanking the two predicted LigR binding sites in Panel B. The pathway on the right illustrates the role of the indicated gene products in *N. aromaticivorans* metabolism of H-, G-, and S-type aromatics. 3-MGA, 3-O-methylgallic acid; CHMOD, 4-carboxy-2-hydroxy-6-methoxy-6-oxohexa-2,4-dienoate; OMA, 4-oxalomesaconate; PDC, 2-pyrone-4,6-dicarboxylic acid.

We previously used DAP-seq (17) to assay binding of 44 predicted *N. aromaticivorans* transcription factors to sequences across the genome *in vitro* (18). Use of this assay showed that a purified *N. aromaticivorans* protein (SARO_RS14285) with 53% amino acid identity and 66% amino acid similarity to *S. lignivoran*s SYK-6 LigR (Fig. S1) showed statistically significant *in vitro* binding to a single region of genomic DNA (indicated by the asterisk in Fig. 1A; Table S1). Higher resolution analysis of this region revealed two statistically significant binding sites for this protein flanking *ligR* and upstream of two aromatic-inducible TSS (14) (Fig. 1B). Most of the genes in these two flanking transcription units (Fig. 1C) encode proteins known to be involved in the 4,5-extradiol branch of PCA catabolism (5, 19). However, two of the genes flanking *ligR* encode subunits of the LigAB1 dioxygenase that is also involved in metabolism of 3-MGA and gallic acid (GA), which are intermediates in SA metabolism (Fig. 1C). Another flanking gene, SARO_RS14280, has no known role in aromatic metabolism, but it is annotated as an NAD(P)-dependent oxidoreductase.

It is interesting to note that the order of genes flanking *ligR* is similar in *N. aromaticivorans* and *S. lignivoran*s SYK-6, but the percent amino acid identity between the proteins predicted to be involved in aromatic metabolism (68–91%) is higher than that between their respective LigR homologs (53%) (Table S2). For comparison, the percent identity of the alpha subunit of RNA polymerase (RpoA), a housekeeping protein, in these two bacteria is 88%. Thus, we refer to *N. aromaticivorans* SARO_RS14285 as LigR given the amino acid sequence conservation, a similar organization, and roles of gene products that flank *ligR*.

### Loss of LigR alters *N. aromaticivorans* metabolism of H-, G-, and S-type aromatics

**Fig 2:**
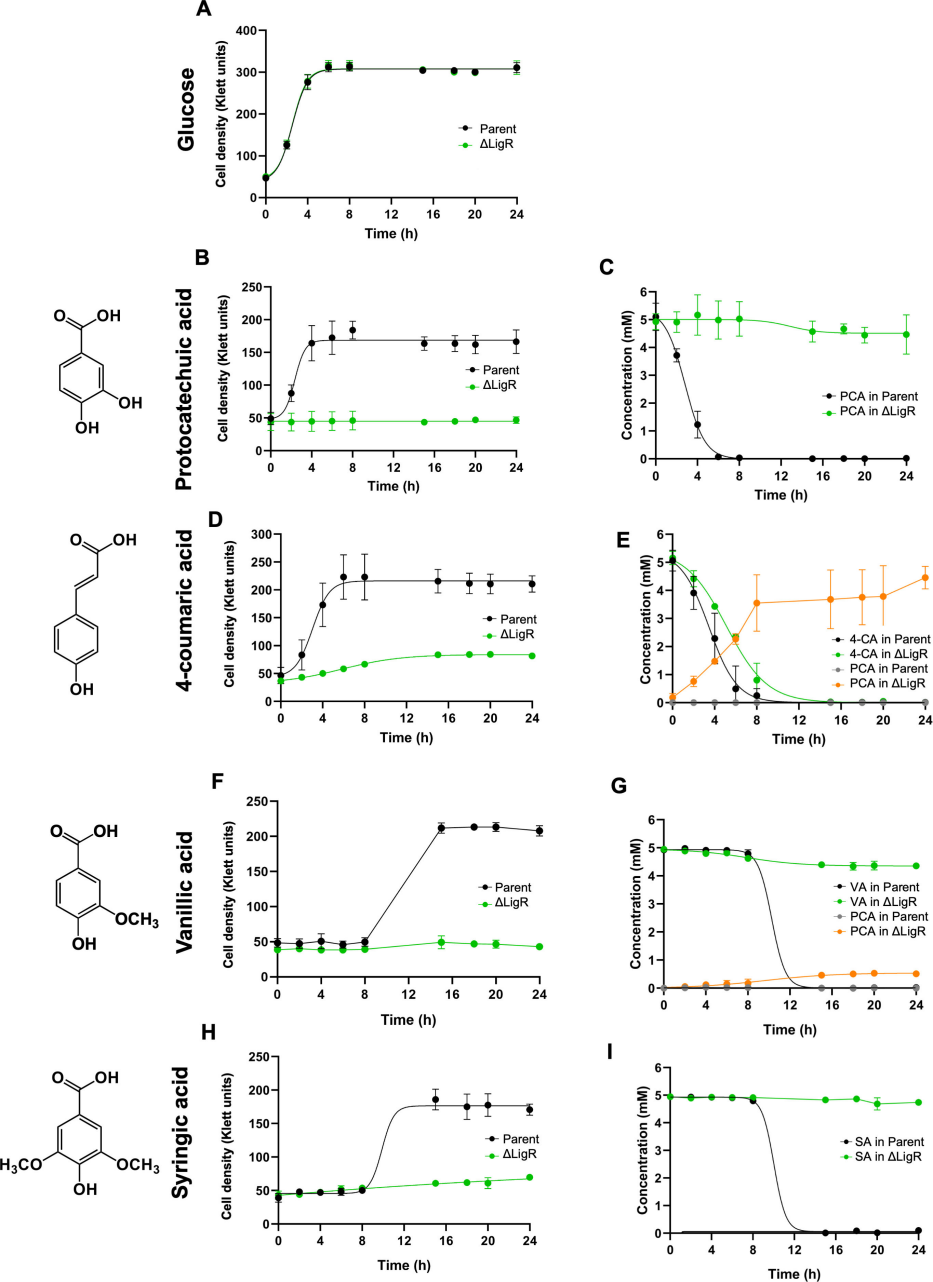
Cell density (**A, B, D, F, and H**) and extracellular concentration of aromatics (**C, E, G, and I**) of the *N. aromaticivorans* parent and ΔLigR mutant grown with glucose (**A**), protocatechuic acid (PCA) (**B and C**), 4-coumaric acid (4-CA) (**D and E**), vanillic acid (VA) (**F and G**), or syringic acid (SA) (**H and I**) as sole carbon sources. Each value is an average ± SD of three replicate cultures.

We used a strain containing an in-frame deletion of *ligR* (termed ΔLigR, Table 1) to test the impact of this mutation on *N. aromaticivorans* metabolism of aromatic carbon sources, and with glucose as a sole non-aromatic carbon source. To do this, we compared the growth of the ΔLigR and parent strains, measured the loss of the aromatic substrate from the culture medium, and quantified the extracellular levels of a set of known intermediates in H-, G-, and S-type aromatic metabolism (5, 12, 19). We found that the ΔLigR mutant grew as well as its parent strain with glucose as a sole carbon source (Fig. 2A), but it was unable to grow with PCA as a sole carbon source (Fig. 2B). We also found that PCA was not removed from the media of the ΔLigR culture, indicating that LigR is required for catabolism of this compound (Fig. 2C).

**TABLE 1:** Strains used in this study

	Genotype	Reference
*Novosphingobium aromaticivorans* strains		
DSM 12444	DSM 12444 ΔSaro_1879	(10)
ΔSARO_RS14285 (ΔLigR)	DSM 12444 ΔSaro_1879 ΔSARO_RS14285	This study
*Escherichia coli* strains		
NEB 5-alpha	*fhuA2Δ(argF-lacZ)U169 phoA glnV44 Φ80Δ(lacZ)M15 gyrA96 recA1 relA1 endA1 thi-1 hsdR17*	New England Biolabs
WM6026	*lacIq, rrnB3, DElacZ4787, hsdR514,* DE(*araBAD*)567*,* DE(*rhaBAD*)568*, rph-1 att-lambda::pAE12-del (oriR6K/cat::frt5), Δ4229(dapA)::*frt(DAP–)*, (endA)::*frt*, uidA(MluI)::pir*(wt)*, attHK::pJK1006::Δ1/2(oriR6Kcat::*frt5*, trfA::frt*)	(20)
Lemo21(DE3)	*fhuA2 [lon] ompT gal (λ DE3) [dcm] ∆hsdS/* pLemo(CamR) *λ DE3 = λ sBamHIo ∆EcoRI-B int::(lacI::PlacUV5::T7 gene1) i21 ∆nin5* pLemo = pACYC184*-PrhaBAD-lysY*	New England Biolabs

PCA is also a key intermediate in the *N. aromaticivorans* pathways for metabolism of the G-type aromatic VA and the H-type aromatic 4-coumaric acid (4-CA) (5). When the ΔLigR mutant was provided 4-CA as a sole carbon source, growth was observed at a slow rate and the final culture cell density was lower compared to the parent strain (Fig. 2D), suggesting there was partial conversion of this aromatic into microbial biomass. Indeed, we observed a slow depletion of 4-CA from the media and nearly stoichiometric accumulation of extracellular PCA by the ΔLigR mutant (Fig. 2E). No other extracellular product of 4-CA metabolism was detected, supporting the hypothesis that the lack of a functional *ligR* gene creates a bottleneck in the normal PCA metabolism, and that the observed growth of this strain was due to removal and subsequent metabolism of an acetyl group from 4-CA.

In addition, we found that the ΔLigR mutant exhibited a strong growth defect, with minimal increase in cell density, when using VA as sole carbon source (Fig. 2F), and that only ~15% of this aromatic was slowly converted to extracellular PCA over the course of the experiment (Fig. 2G). We propose that limited activity of the *N. aromaticivorans* aromatic demethylases (DesA and LigM; Fig. 1C) (5) can explain why only a fraction of the VA was metabolized to PCA by the ΔLigR mutant.

*N. aromaticivorans* can use one of two pathways to catabolize the S-type aromatic SA (19), with 3-MGA as a common initial intermediate that results from SA demethylation by DesA (Fig. 1C). One route uses the LigAB1 dioxygenase, encoded by genes downstream of *ligR*, for ring opening of 3-MGA (Fig. 1C). The other route uses LigM or DmtS for demethylation of 3-MGA to GA before LigAB1 or LigAB2 catalyze GA ring opening (19). We found that the ΔLigR mutant had a strong growth defect when using SA as a sole carbon source (Fig. 2H) and did not significantly reduce extracellular levels of this aromatic carbon source (Fig. 2I). In addition, there was no accumulation of detectable levels of known pathway intermediates over the course of the experiment (Fig. 2I), indicating that neither route was functional. Both routes for SA metabolism require tetrahydrofolate-dependent demethylases (19), so we propose that, similar to what was observed for VA metabolism by the ΔLigR mutant, limited aromatic demethylation may explain the behavior of the △LigR mutant when provided with SA as the sole carbon source.

### LigR increases expression of several aromatic metabolism genes

To evaluate the effect of LigR on the expression of genes involved in aromatic metabolism, we used RNA-seq to measure genome-wide transcript levels in the parent strain and the ΔLigR mutant. However, since the ΔLigR mutant exhibited growth defects when tested with several aromatics as sole organic carbon sources (Fig. 2), we compared transcript levels when the parent strain and the ΔLigR mutant were grown in medium with glucose alone, or with glucose plus either PCA, VA, 4-CA, or SA (21). This approach was based on previous work showing that transcripts for many of the *N. aromaticivorans* genes involved in aromatic metabolism are present in higher abundance in cultures containing both glucose and either PCA or VA as carbon sources (9, 22).

**Fig 3:**
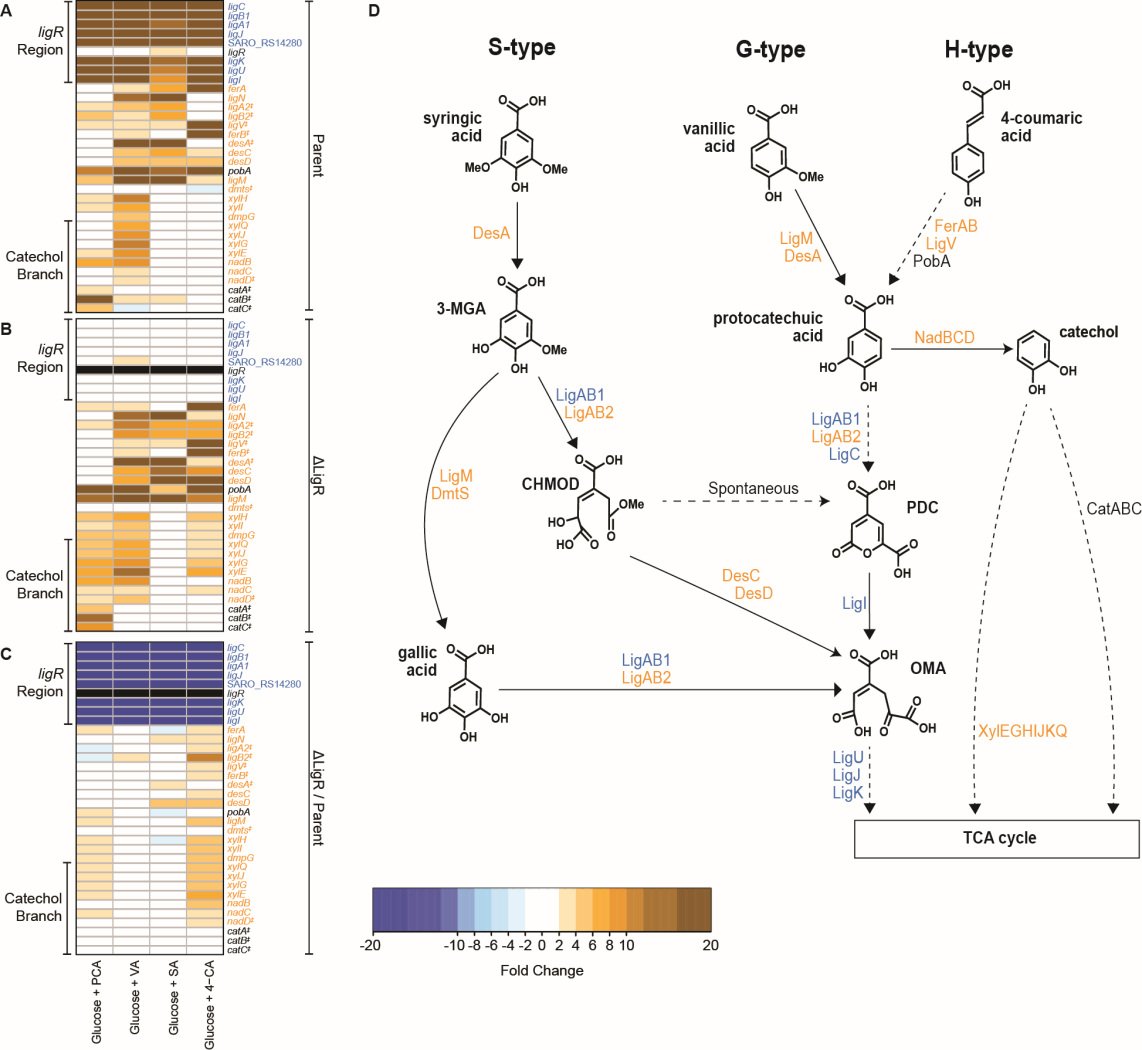
Transcript analysis of the ΔLigR mutant and its parent strain grown in the media containing aromatics with glucose or glucose alone. The heatmaps show transcript levels for genes known to be involved in aromatic metabolism when either the parent strain (**A**) or the ΔLigR mutant (**B**) was grown in media containing glucose and the indicated aromatic compared to cells grown in media where glucose is the sole carbon source. Panel **C** shows the changes in transcript abundance when comparing those in the ΔLigR mutant to those of parent cells grown in the presence of glucose plus the aromatic compound. Shown in orange or blue are genes with a transcript level fold change higher than 2 or lower than −2, respectively, and a false discovery rate (FDR) lower than 0.05. Transcript abundances from genes labeled with an “‡” do not have an FDR lower than 0.05, but they were included in the figure because they are known to be involved in *N. aromaticivorans* aromatic metabolism. Black boxes indicate the lack of detectable transcripts from the *ligR* gene in the ΔLigR mutant. Panel **D**: Summary of aromatic metabolism in *N. aromaticivorans*, with enzyme names color-coded to reflect the changes in transcript abundance indicated in Panel **C**. Dashed arrows indicate more than one reaction in the metabolic conversion. Complete transcript abundance data sets for each sample are in Fig. S2 to S4 and Table S3 (21). 3-MGA, 3-O-methylgallic acid; CHMOD, 4-carboxy-2-hydroxy-6-methoxy-6-oxohexa-2,4-dienoate; OMA, 4-oxalomesaconate; PDC, 2-pyrone-4,6-dicarboxylic acid.

We found statistically significant changes in transcript levels when comparing the parent strain grown in either glucose alone or glucose and one of the aromatics tested (Table S3). For example, the expression of genes known to be involved in the metabolism of PCA, VA, 4-CA, or SA was higher when the parent strain was grown in the presence of both glucose and one of these aromatics than when the media contained glucose as a sole carbon source (Fig. 3A; Table S3). Specifically, mRNA levels from genes in the two transcription units that flank the *ligR* gene, termed *ligR* region in Fig. 3A through C, were ~5- to 20-fold more abundant when the parent strain was grown in media containing glucose and either PCA, VA, SA, or 4-CA, as compared to cultures utilizing glucose as the sole carbon source (Fig. 3A). These increases in transcript levels from the l*igR* region agree with prior observations when PCA or VA were used as the aromatic substrate (22). In addition, aromatic-inducible genes were also found outside the *ligR* region in the parent strain (Fig. 3A and D). These other aromatic-inducible genes included ones known to be involved in the conversion of 4-CA or VA to PCA, the assimilation of PCA, or those known or predicted to participate in conversion of SA to CHMOD (5, 19). Other transcripts that were present at increased abundance when the parent is grown in the presence of VA or SA included those encoding the DesA and LigM demethylases.

Analysis of the ΔLigR mutant grown under the same set of conditions revealed that this protein was required for increased expression of the aromatic-inducible genes flanking *ligR*. As expected from the deletion of this gene, the ΔLigR mutant lacked detectable *ligR*-specific transcripts in cultures grown in the presence of glucose or glucose and any of the aromatics tested (Fig. 3B). In addition, transcripts from flanking genes in the *ligR* region (*ligCB1A1J -* SARO_RS14280 and *ligKUI*) were no longer inducible in the ΔLigR mutant (Fig. 3B) since mRNA levels did not increase when this strain was grown in the presence of PCA, VA, SA, or 4-CA and glucose. From these results, we propose that LigR is required to increase expression of flanking genes in the *ligR* region when cells are grown with the H-, G-, and S-type aromatics used in this work (PCA, 4-CA, VA, or SA). This flanking region encodes the genes involved in the 4,5-extradiol branch of PCA catabolism (Fig. 3D).

### Loss of LigR results in use of alternative aromatic pathways

Our RNA-seq analysis also revealed a significant, but unexpected, increase in the expression of genes coding for enzymes known or predicted to be involved in alternative routes for metabolism of H-, G-, or S-type aromatics in the ΔLigR mutant compared to the parent strain (Fig. 3C). Transcripts with statistically significant increased levels when the ΔLigR mutant was grown in the presence of the 4-CA plus glucose compared to the parent strain included those encoding the LigM demethylase (>5-fold) that generates PCA from VA, during metabolism of G-type aromatics, and GA from 3-MGA, during metabolism of S-type aromatics, and LigAB2 (~3- to 5-fold), a second dioxygenase that catalyzes ring opening of PCA, 3-MGA, and GA (19). In addition, *nadBCD* transcripts, encoding proteins needed for decarboxylation of PCA to catechol (12), were >5-fold higher when the ΔLigR mutant was grown in the presence of glucose and 4-CA than in the parent strain grown under the same conditions (Fig. 3C). The abundance of *xylEGHIJKQ* transcripts, which encode enzymes that participate in the extradiol pathway for catechol degradation, was also increased ~3- to 5-fold in the ΔLigR mutant compared to the parent strain when cells were grown in the presence of 4-CA or PCA and glucose (Fig. 3C). We also found that numerous genes known or predicted to be involved in glucose metabolism or function of non-aromatic pathways showed changes in expression in one or more cultures (Fig. S2 to S4). Overall, the transcript abundance differences between the ΔLigR mutant and the parent strain reveal both common and variable gene expression changes depending on the aromatic added to the growth media, suggesting the existence of additional LigR-independent but aromatic-dependent branches of a transcriptional network.

**Fig 4:**
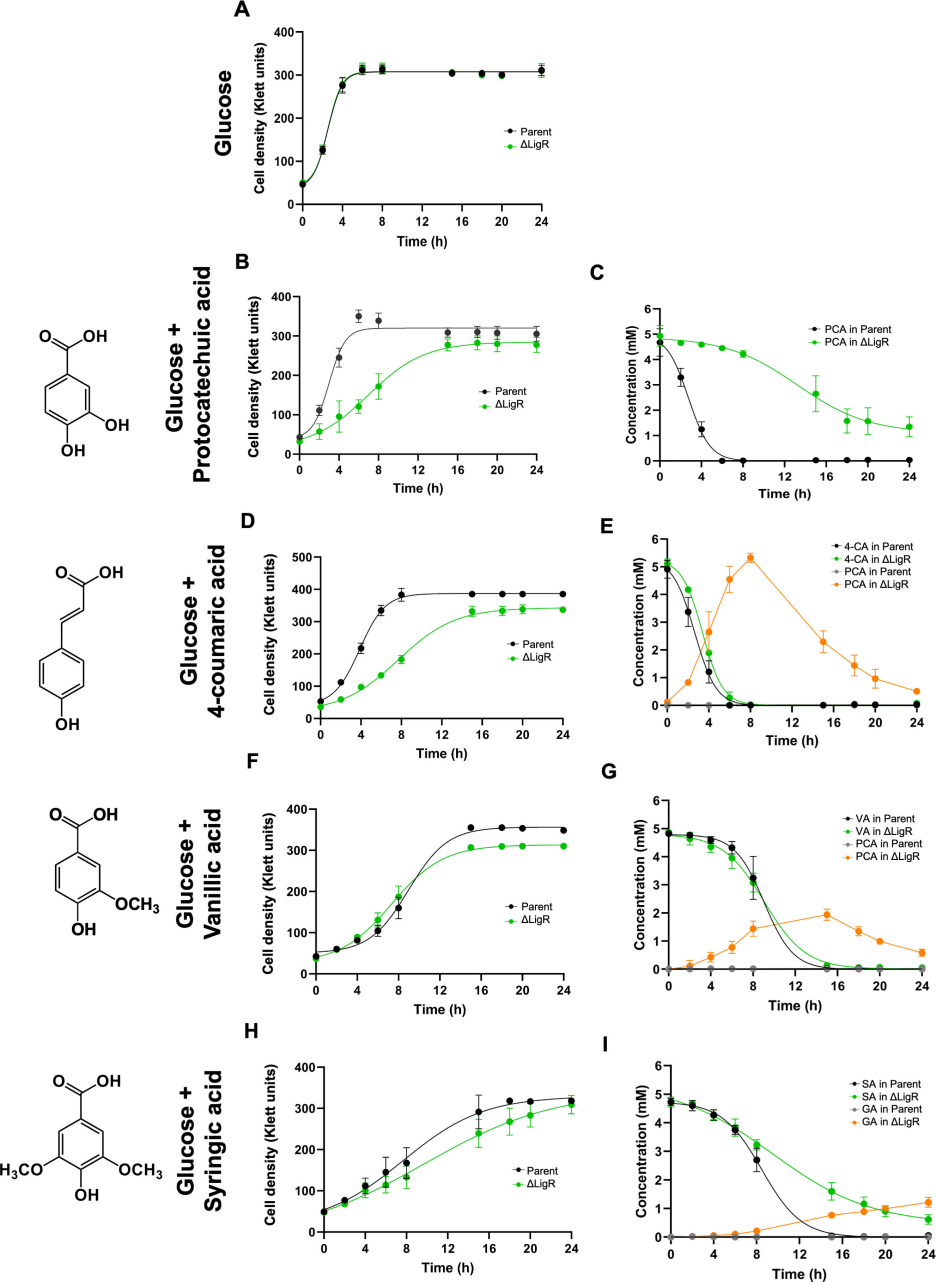
Growth (**A, B, D, F, and H**) and extracellular aromatic concentrations (**C, E, G, and I**) of the ΔLigR mutant and the parent strain in media containing glucose alone (**A**), or the aromatics protocatechuic acid (PCA) (**B and C**), 4-coumaric acid (4-CA) (**D and E**), vanillic acid (VA) (**F and G**) or syringic acid (SA) (**H and I**) and glucose. The extracellular intermediates in aromatic metabolism are shown (**B, D, F, and H**). Each value is an average ± SD of three replicate cultures. GA, gallic acid.

To test if aromatics were these differences in gene expression led to rerouting of aromatic metabolism in the ΔLigR mutant, we compared the extracellular concentrations of a set of known pathway intermediates in the ΔLigR mutant and the parent strain when they were grown in the presence of glucose and single aromatics. We chose this approach since previous studies have shown that wild-type and mutant strains of *N. aromaticivorans* are capable of co-metabolism of glucose and aromatic compounds (5, 19). In media containing glucose and each of the aromatics tested, both the ΔLigR and parent strains were able to grow, metabolize the glucose, and achieve comparable cell densities (Fig. 4B, D, F and H). By quantifying extracellular concentrations of a targeted set of metabolites, we found that the ΔLigR mutant could more efficiently remove each of the aromatics from the media (Fig. 4C, E, G and I) when glucose was present, compared to the behavior observed when the mutant was cultured in media containing only aromatics (Fig. 2).

For example, when cultures were provided PCA and glucose, we observed metabolism of the aromatic by the ΔLigR mutant, albeit at a slower rate and to a lower extent than what is found in the parent strain (Fig. 4B and C). By the end of the experiment, ~27% of the initial PCA remained in the media of the ΔLigR mutant, likely explaining why the mutant culture reached a lower final cell density than the parent strain. No other extracellular metabolites accumulated to detectable levels in this culture. In cells grown with glucose and either 4-CA or VA, we found that the aromatic disappeared from the media at similar rates in the ΔLigR mutant and the parent strain, but PCA accumulated transiently in the media of the mutant grown in the presence of either aromatic (Fig. 4E and G). The lower abundance of *ligAB1* transcripts in the ΔLigR mutant (Fig. 3B) and the transient accumulation of PCA in cultures provided with glucose and either 4-CA or VA mimics accumulation of this pathway intermediate when an *N. aromaticivorans* mutant lacking LigAB1/2 activity grows in media containing VA and glucose (12). In addition, when the ΔLigR mutant was grown in media containing SA and glucose, we observed incomplete metabolism of the aromatic and extracellular accumulation of GA (Fig. 4I), an intermediate in the conversion of SA to OMA (Fig. 3D). Because in the ΔLigR mutant, the metabolism of OMA is restricted, but aromatic transformation is still observed, we propose that the growth of the ΔLigR mutant in the presence of glucose and SA alters the route by which pathway intermediates are metabolized.

### LigR binds to aromatic metabolism gene promoters

**Fig 5:**
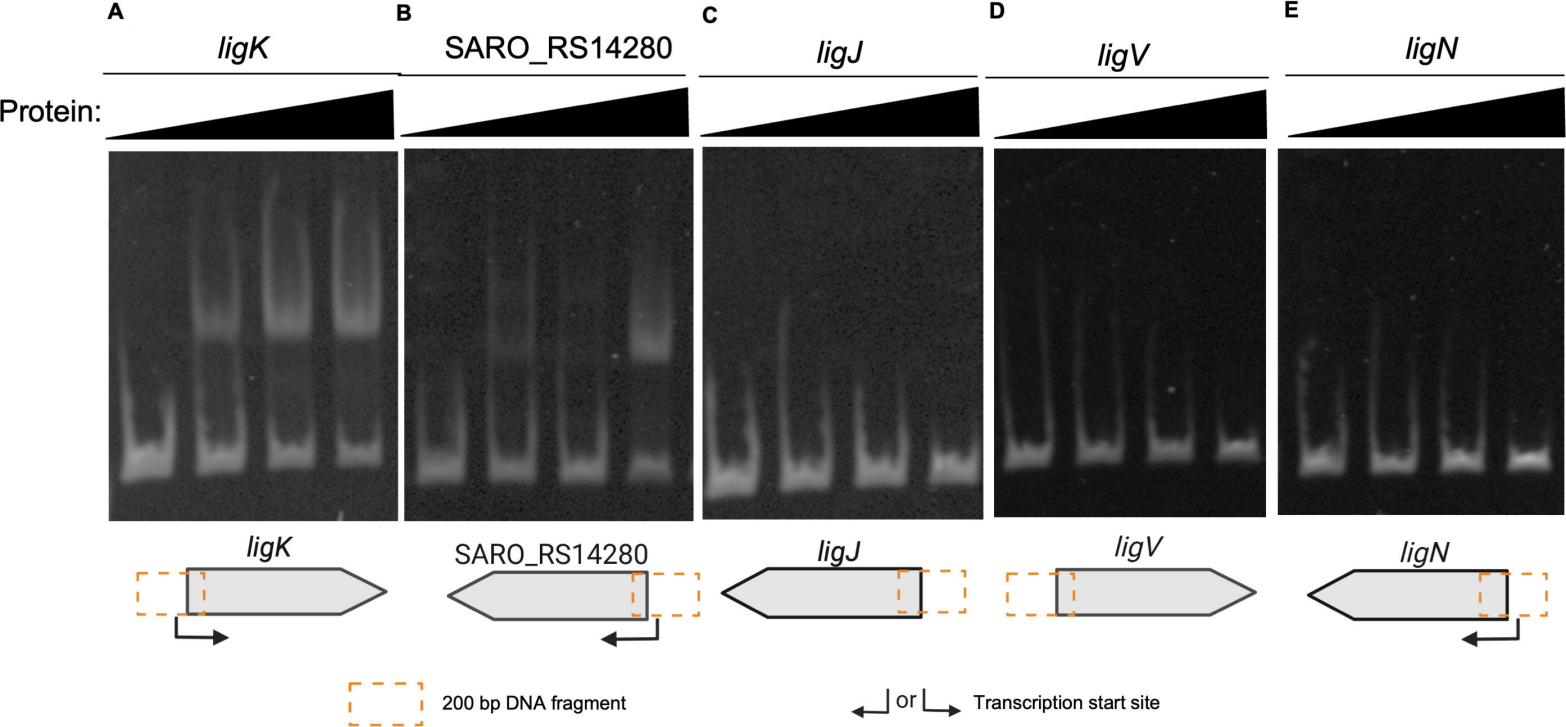
Electrophoretic mobility shift assay for DNA binding by recombinant LigR. Each assay examined binding to a ~200 bp sequence in the indicated region of genes *ligK* (**A**), SARO_RS14280 (**B**), *ligJ* (**C**), *ligV* (**D**), and *ligN* (**E**). To each reaction, 0, 100, 300, or 500 nM of the recombinant LigR protein was added.

The DAP-seq and RNA-seq analyses support the hypothesis that LigR directly regulates the expression of genes required for the catabolism of the H-, G-, or S-type aromatics tested. To test this hypothesis, we used an electrophoretic mobility shift assay to evaluate LigR binding to DNA sequences upstream of several genes with altered transcript levels when the ΔLigR mutant is grown in the presence of glucose and one of the H-, G-, or S-type aromatics tested (Fig. 5). When the purified LigR protein (Fig. S5) was incubated with DNA encompassing 200 bp flanking the TSS for the *ligKUI* or the SARO_RS14280 – *ligJA1B1C* regions (14), we observed a protein-dependent shift in their electrophoretic mobility (Fig. 5A and B). Under identical conditions, we did not observe a shift in the electrophoretic mobility of similarly-sized DNA fragments upstream of *ligJ*, *ligN*, and *ligV* (Fig. 5C through E), regions that did not show statistically significant binding to LigR in the DAP-seq assay (Fig. 1). The failure of purified LigR to shift mobility of *ligV* (involved in 4-CA metabolism) or *ligN* (β-O-4 aromatic dimer metabolism) DNA fragment (10) leads us to propose that the increased abundance of transcripts from their respective genes in the ΔLigR mutant cultured in 4-CA plus glucose (Fig. 3D) does not reflect direct repression of these genes by LigR.

**Fig 6:**
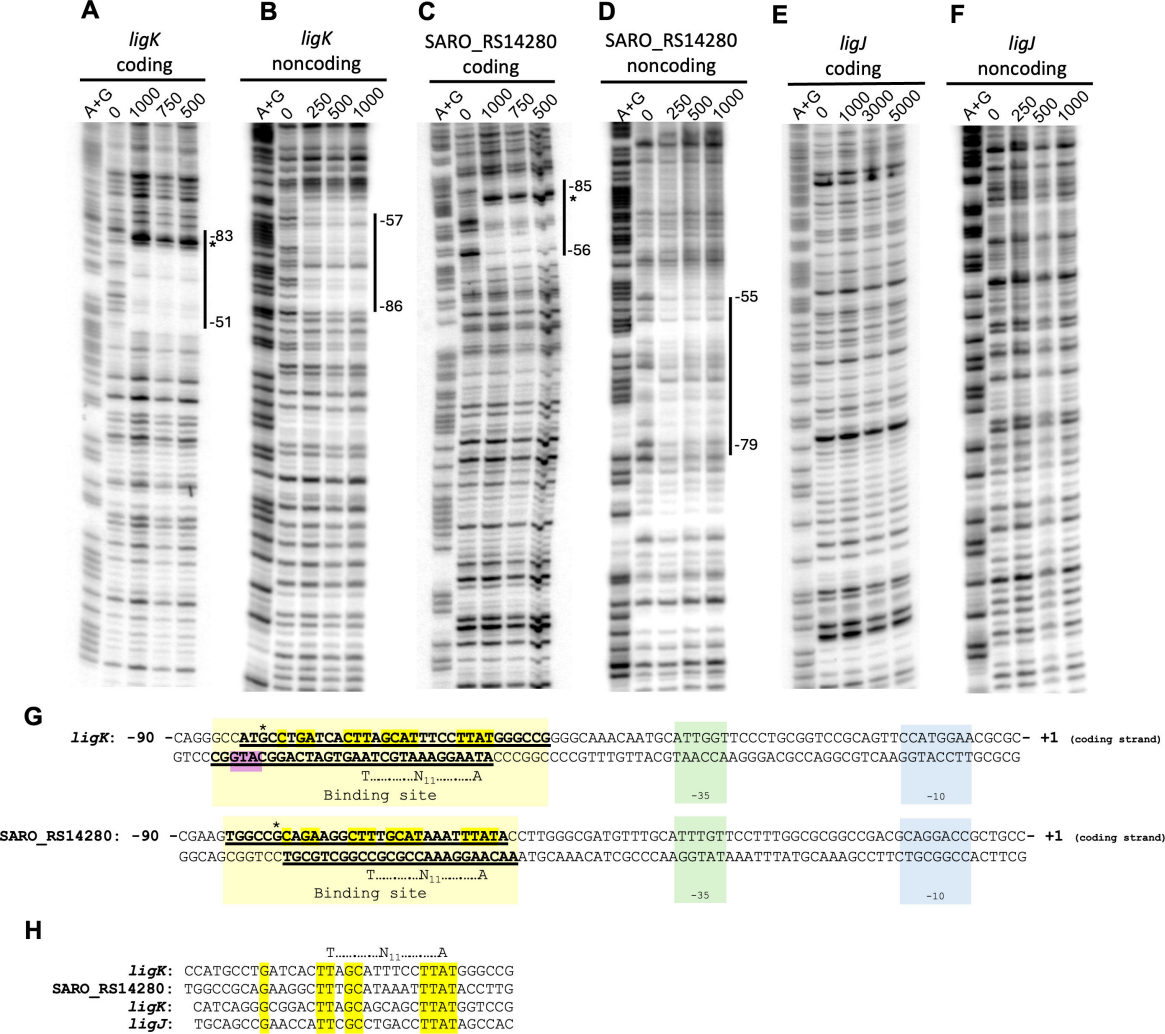
DNase I protection of SARO_RS14280 and *ligK* promoter regions by recombinant LigR. Either the 5′-ends of the coding or noncoding strands for the promoter regions of *ligK* (**A and B**), SARO_RS14280 (**C and D**), and *ligJ* (**E and F**) were radiolabeled before treating with DNase I in the absence or presence of the indicated amount of LigR protein (nM). To map the binding sequences, samples were electrophoresed alongside Maxam-Gilbert (A + G) DNA sequencing ladders. An asterisk indicates a DNase I hypersensitive site in assays that contain recombinant LigR. Panel **G** shows DNA sequences 90 bp upstream of the transcription start sites (14) for *ligK* and SARO_RS14280. Blue, green, and yellow boxes indicate the −10 element, the −35 element, and the LigR binding sites for *ligK* and SARO_RS14280, respectively. Highlighted in yellow is a conserved DNA sequence in the LigR binding sites of these two promoter regions. The purple box shows the proposed start codon (ATG) for l*igR* translation. The –10 and –35 elements of *ligR* are not shown because the TSS has not been determined (see text). Panel **H** shows an alignment of the binding sites for *N. aromaticivorans* LigR upstream of *ligK* and SARO_RS14280 (top) and for *S. lignivoran*s SYK-6 LigR upstream of *ligK* and *ligJ*. Highlighted in yellow are nucleotides that are conserved in these four binding sites.

To define the LigR DNA-binding sequence, we used DNase I footprinting reactions. We found that purified LigR protected ~30 bp in the *ligK* (Fig. 6A and B) and SARO_RS14280 (Fig. 6C and D) promoter regions from DNase I cleavage. The DNA sequence protected from DNase I digestion in both DNA fragments lies ~50–85 nucleotides upstream of the TSS for these genes. The *ligK* region protected by LigR is also close to the divergently transcribed *ligR* gene (Fig. 6).

LigR binding to the *ligK* and SARO_RS14280 DNA fragments also results in an enhancement of DNase I digestion on the coding strand at the upstream end of the protected region (indicated by the asterisk in Fig. 6), suggesting that protein binding alters the DNA conformation and makes the fragment more susceptible to enzyme digestion. Under identical conditions, LigR failed to protect a *ligJ* promoter fragment from DNAse I, in agreement with the lack of this protein to alter migration of the same DNA fragment in the electrophoretic mobility shift assay (Fig. 5C). Thus, the combined results from the DAP-seq, electrophoretic mobility shift, and DNase I footprinting assays lead us to propose that LigR directly activates transcription of the *ligK* and the SARO_RS14280 operons by binding specific sequences upstream of their respective promoters.

### Conservation of *ligR* and its flanking target genes in other bacteria

Given the key role of *N. aromaticivorans* LigR in an aromatic-inducible transcriptional network, we asked whether this transcription factor and its direct target genes are co-localized in other bacteria. We first screened bacterial genomes for the presence of *ligR* and the *ligCB1A1J*–SARO_RS14280 or *ligKUI* transcription units. A query of 4,826 reference bacterial genomes identified 29 organisms that met these criteria. Of these, 25 are Alphaproteobacteria and 4 are Gammaproteobacteria (*Gynuella sunshinyii* YC6258, *Saccharospirillum mangrovi, Vibrio fortis,* and *Vibrio natriegens* ATCC 14048). Of the Alphaproteobacteria identified in this query, 20 belong to the order Sphingomonadales (Table S4).

**Fig 7:**
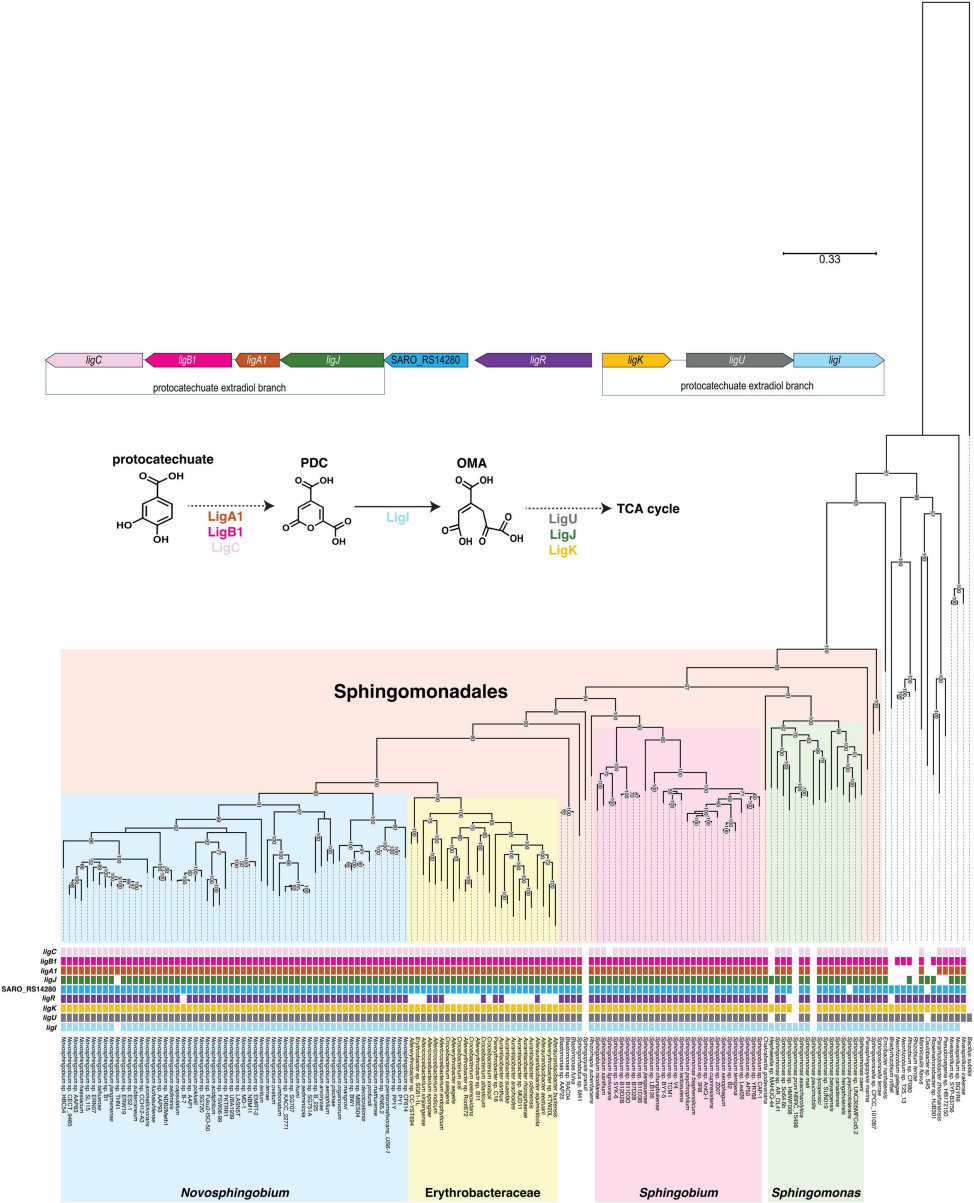
Phylogenetic analysis of genes surrounding the *ligR* gene across 152 bacterial genomes. The phylogenetic tree was adapted from an analysis made by Metz et al. (9). Each gene is color-coded in its genomic context, the aromatic degradation pathway, and the phylogenetic tree. This figure shows if the genes studied are present or not in each genome. Genomic position data were used to examine if these genes are in the same organization as in *N. aromaticivorans* DSM 12444 (Table S4). OMA, 4-oxalomesaconate; PDC: 2-pyrone-4,6-dicarboxylic acid.

We then queried a set of 152 genome sequences of known aromatic metabolizing bacteria, including 137 Sphingomonadales and *Pseudomonas putida,* an organism often studied for this metabolic activity (9). We found that 125 genomes (~82%) possess a *ligR* homolog, while 113 of these (~74% of the total) possess both a *ligR* gene and predicted *ligCB1A1J*–SARO_RS14280 and *ligKUI* transcription units. Of these 113, 82 contain a *ligR* gene flanked by predicted *ligCB1A1J*–SARO_RS14280 and *ligKUI* transcription units as is found in *N. aromaticivorans* DSM 12444 (Fig. 7; Table S4). All 82 of these bacteria belong to the order Sphingomonadales, with 77 in the family Sphingomonadaceae and 5 in the family Erythrobacteraceae. These results predict that the co-localization of *ligR* and its flanking target genes (*ligCB1A1J*–SARO_RS14280 and *ligKUI*) is mostly limited to the family Sphingomonadaceae.

## DISCUSSION

*N. aromaticivorans* DSM 12444 can use several routes to convert natural and chemically modified aromatics into central carbon intermediates, which are normally used to support growth (5, 12, 19). These aromatic metabolic pathways have recently been engineered to create strains that funnel one or multiple aromatics into potential industrial products (9, 12, 13). While enzymes involved in the synthesis or use of the intermediates have been identified (5, 19), less is known about systems that control these aromatic-inducible pathways. This work combined transcriptomics, targeted metabolite analyses, genomics, and *in vitro* studies to gain a systems-level understanding of how *N. aromaticivorans* regulates the use of different routes for aromatic metabolism.

### LigR is a transcriptional activator of genes involved in multiple aromatic pathways

Our results lead us to conclude that LigR is a direct transcriptional activator of a set of genes required for metabolism of multiple types of aromatics. The ΔLigR mutant exhibited defects in growth or metabolism of several types of aromatics, and it showed a ~10- to 20-fold decrease in expression of genes flanking *ligR* compared to a parent strain when grown in the presence of PCA, 4-CA, VA, or SA and glucose. Several *in vitro* assays showed that LigR bound sequences upstream of the *ligK* and SARO_RS14280 TSS. The position of this LigR binding site, between −51 to −83 and −56 to −85 bp relative to the *ligK* and SARO_RS14280 TSS, respectively, is similar to that of class I bacterial transcriptional activators that bind upstream of the −35 promoter element (23, 24), including several other LysR-family transcriptional activators and *S. lignivoran*s SYK-6 LigR (15, 25–27). While primer extension assays have mapped the 5′ ends of LigR-dependent genes in *S. lignivoran*s SYK-6, their TSS have not been determined. However, we note that the SYK-6 LigR binding sites were located at a similar position (−48 to −80 bp) (15) as those for the *N. aromaticivorans* protein (−51 to −85 bp).

The regions protected from DNase I digestion by *N. aromaticivorans* and *S. lignivoran*s SYK-6 LigR homologs contain a conserved TT-N-GC-N_6_-TTAT sequence (Fig. 6H), that overlaps a proposed T-N_11_-A consensus recognition element for LysR-family transcription factors (25, 27, 28). However, these DNA sequence elements are not found upstream of *N. aromaticivorans ligJ*, consistent with the failure to detect LigR binding to this region *in vitro*. LigR binding resulted in a hypersensitive site for DNase I digestion ~80 bp upstream of the TSS of both the *ligK* and SARO_RS14280 genes that could reflect a protein-induced conformational change in the upstream promoter region leading to increased transcription, as is found with other transcription factors (29).

From *in vitro* analysis of *S. lignivoran*s SYK-6 LigR, it is known that the presence of PCA or GA extends the downstream edge of the DNase I footprint created by this protein (15), suggesting these intermediates might alter the interaction of LigR with DNA and its ability to activate transcription by RpoA. However, both *N. aromaticivorans* and *S. lignivoran*s SYK-6 LigR bind DNA upstream of target genes in the absence of any added ligand *in vitro* (15). Thus, it is possible that DNA binding by LigR homologs is not sufficient for transcriptional activation and may require a ligand for stimulating transcription from promoters.

While LigR might require a ligand to increase transcription, the abundance of *ligR* transcripts was ~20- to 100-fold lower than that of the flanking genes and there was no statistically significant change in abundance of this mRNA when the parent strain was grown in the presence of glucose plus PCA, 4-CA, VA, or SA compared to growth in glucose alone, or in published data from cells grown with other aromatic monomers or dimers (9, 22). We have been unable to map a TSS for *ligR* using RNA from cells grown in the presence of either glucose or VA (14), and the low expression of this gene has made it difficult to predict the location and sequence of the *ligR* promoter. If transcript levels are taken as a predictor of protein abundance, it suggests that LigR levels are low when cells are grown both in the absence and presence of aromatics tested in this and previous studies.

### LigR impacts use of several aromatic metabolic pathways

The *N. aromaticivorans* genome encodes enzymes to metabolize H-, G-, and S-type aromatic monomers, converting them into TCA cycle intermediates that are normally used to support growth (5, 19, 22). The capability to metabolize all three aromatic types is not always found in other aromatic metabolizing bacteria, so strains are often engineered to expand the suite of substrates that can be metabolized or to funnel intermediates into potential industrial products that cannot be made by wild-type strains. While *S. lignivoran*s SYK-6 and *N. aromaticivorans* can both metabolize multiple aromatic types, there are differences in the role of LigR in these two bacteria. For example, the *N. aromaticivorans* ΔLigR mutant exhibits a growth defect in metabolism of PCA, 4-CA, VA, or SA as a sole carbon source. In contrast, a *S. lignivoran*s SYK-6 LigR mutant exhibits only a slight growth defect when provided with VA or SA as a sole carbon source (16).

**Fig 8:**
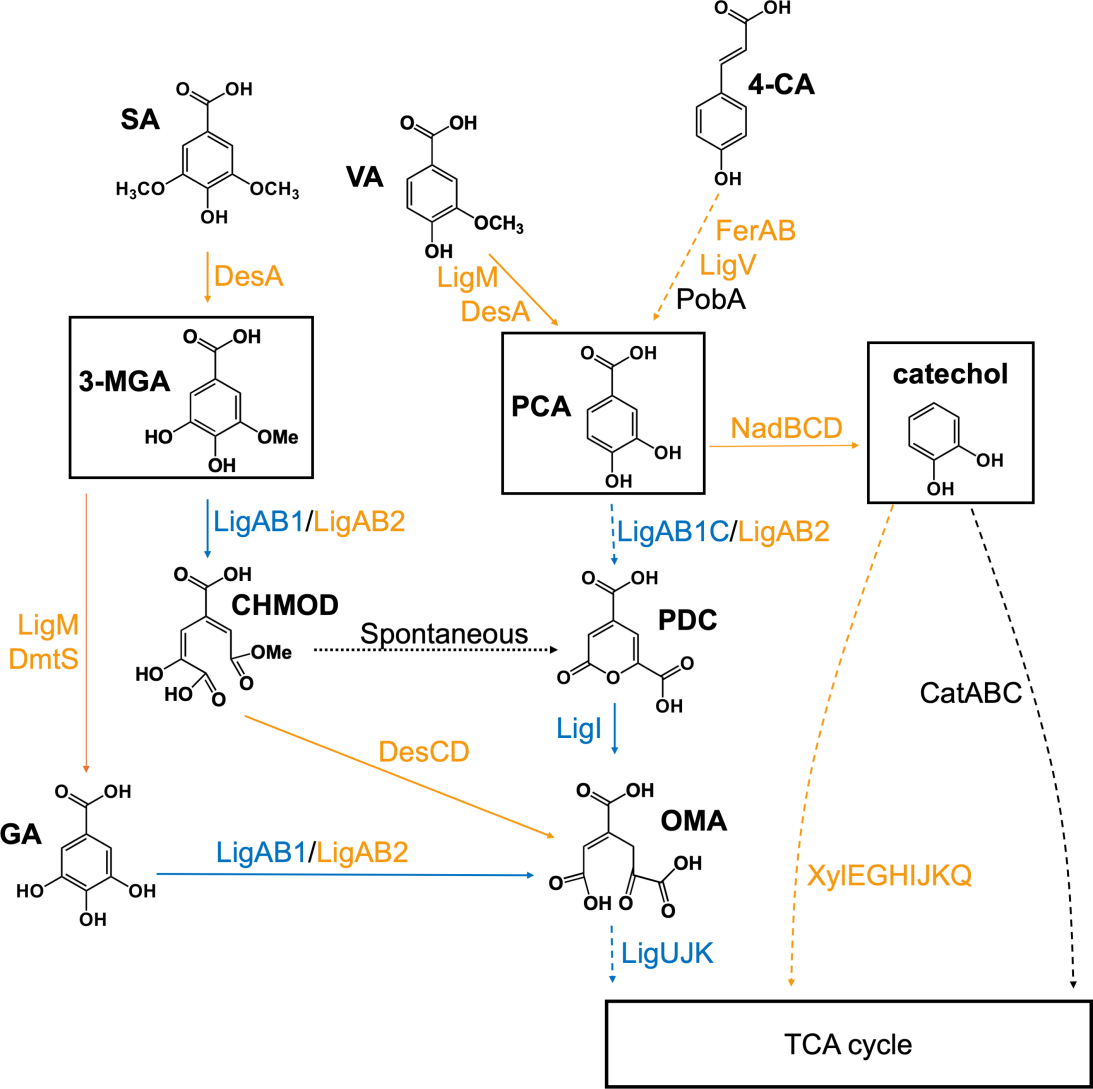
Metabolic nodes influenced by the transcription factor LigR. Blue arrows indicate the reactions activated by LigR in the parent strain. Yellow arrows show alternative pathways favored in the ΔLigR mutant. The intradiol branch for catechol degradation, which is not affected by the presence or absence of LigR, is shown by a solid black arrow. The dotted black arrow indicates a spontaneous reaction. 3-MGA, 3-O-methylgallic acid; 4-CA, 4-coumaric acid; CHMOD, 4-carboxy-2-hydroxy-6-methoxy-6-oxohexa-2,4-dienoate; GA, gallic acid; OMA, 4-oxalomesaconate; PCA, protocatechuic acid; PDC, 2-pyrone-4,6-dicarboxylic acid; SA, syringic acid; VA, vanillic acid.

This work provides new insight into the *in vivo* role of *N. aromaticivorans* LigR in metabolism of different aromatic types. The metabolic fate of intermediates when cells grow in representative G- (VA), H- (4-CA), and S- (3-MGA) type aromatics, and in the intermediate PCA depends on the presence of LigR (Fig. 8). Indeed, our analysis of *N. aromaticivorans* LigR function provides direct evidence for the role of this protein in using 3-MGA, PCA, and catechol as critical intermediates in the aromatic metabolic network of this bacterium (5, 12, 13). 3-MGA is an intermediate in two potential pathways for *N. aromaticivorans* SA metabolism (Fig. 8). In wild-type cells, the combined activity of the LigAB1 and LigAB2 dioxygenases converts ~85% of the 3-MGA to CHMOD, while a combination of LigM and DmtS converts ~15% of the 3-MGA to GA (19). In contrast, when the ΔLigR mutant is grown in media containing SA and glucose, we propose that the majority of the 3-MGA produced is diverted to GA by LigM and DmtS since we observed GA but not PDC accumulation and since there is low-level expression of genes that encode enzymes that divert these intermediates to TCA cycle (Fig. 8). Since the accumulated GA was not stoichiometric to the metabolized SA, it is also possible that the absence of a functional *ligR* gene reroutes intermediates of SA metabolism to as of yet unidentified catabolic pathways.

Our data show that there are also different metabolic fates for PCA in the parent and the ΔLigR mutant strains (Fig. 8). Wild-type cells route all of the PCA *via* the 4,5-extradiol catabolic pathway into PDC, either when grown in PCA as a sole carbon source or when it is generated by metabolism of 4-CA and VA (5, 12). While the *N. aromaticivorans* genome encodes a PCA decarboxylase NadCD, decarboxylation of this compound to catechol is not a major metabolic route in wild-type cells grown in PCA, 4-CA, or VA in the presence or absence of glucose (5, 12, 19). Indeed, in previous work, conversion of PCA to catechol required placing *nadCD* under control of the *ligAB1* promoter, which we show here is LigR-dependent (12). In this study, increased expression of genes required for PCA decarboxylation (*nadBCD*) and catechol metabolism *via* the extradiol pathway (*xylEGHIJKQ*) was observed when the ΔLigR strain was grown in media containing glucose and PCA or 4-CA. In contrast, *catABC* transcripts, which encode enzymes in the intradiol branch for catechol degradation (12), did not show a statistically significant expression change when the ΔLigR mutant was grown in media containing glucose and either PCA, 4-CA, or VA. Combined, these findings lead us to propose that in *N. aromaticivorans*, PCA is decarboxylated to catechol and is metabolized by the *xylEGHIJKQ*-dependent catechol extradiol pathway when the ΔLigR mutant is grown in media containing glucose and PCA or 4-CA. To our knowledge, this rerouting of PCA to catechol in a ΔLigR mutant has not been reported in other aromatic metabolizing bacteria. We propose that it could be an indirect effect of the *ligR* deletion since binding of LigR to the *nadBCD* or *xylEGHIJKQ* promoters was not detected in the DAP-seq assay (18).

It is interesting to find that aromatics are metabolized by the ΔLigR mutant in the presence of glucose (Fig. 4), but it is prevented or limited in the absence of glucose (Fig. 2). The published RNA-seq data (21) shows there is increased expression of dozens of potential transcription factors when the *N. aromaticivorans* ΔLigR mutant is grown in the presence of glucose and the aromatics tested in this study. To our knowledge, no similar global gene expression analysis has been published in △LigR mutants of other aromatic metabolizing bacteria. Based on these observations, we propose that *N. aromaticivorans* contains other, as of yet, unidentified regulators that alter the route of intermediates through branches in the known aromatic metabolic network (Fig. 8) and that there may be other unknown intermediates in aromatic metabolism that did not accumulate to detectable levels in the media. Thus, additional experiments should be performed to identify the mechanisms that regulate the flow of 3-MGA to known (CHMOD, PDC, and OMA) or possibly unknown pathway intermediates that were not accumulated to detectable levels, direct conversion of PCA to catechol, or route catechol metabolism through extradiol or intradiol pathways. *N. aromaticivorans* is known or predicted to metabolize a variety of sugars, organic acids, and other compounds as carbon sources (6, 7, 22), so future studies also need to determine if this rerouting of intermediates through branches of the aromatic metabolic network occurs when the ΔLigR mutant or a wild-type strain is grown in media containing any of these other carbon sources.

In summary, our results show that *N. aromaticivorans* LigR is a transcriptional activator of genes needed for utilization of diverse aromatics by a network consisting of multiple branches. This genomic and bioinformatic analysis of LigR provided new systems-level information on the transcriptional control of genes that function in different aromatic branches of this and possibly other bacteria. In addition, we propose that the ability of the ΔLigR mutant to catabolize H-, G-, and S-aromatics *via* pathways different than those used in wild-type cells reveals that intermediates represent key nodes that connect branches of the *N. aromaticivorans* aromatic network. We further propose that future systems-level understanding of the elements and control of bacterial aromatic networks that could help improve the conversion of lignin-derived and other aromatic compounds into products of biotechnological, environmental, and industrial utility.

## MATERIALS AND METHODS

### Bacterial strains, growth media, and culture conditions

*N. aromaticivorans* DSM 12444 Δ1879 (10), a derivative of *N. aromaticivorans* DSM 12444 that lacks the *sacB* gene (SARO_RS09410, formerly Saro_1879), was used as the parent strain in this study. All bacterial strains used in this study are listed in Table 1. Primers are listed in Table S5.

*Escherichia coli* strains were grown at 37°C in LB media or LB supplemented with 0.3 mM diaminopimelic acid when necessary. Antibiotics were added in accordance with the strain resistance (50 µg/mL kanamycin, 30 µg/mL chloramphenicol, and/or 30 µg/mL ampicillin). *N. aromaticivorans* strains were grown at 30°C in standard mineral base (SMB) minimal media (10), supplemented with 10 mM glucose and/or 5 mM aromatic when indicated. All aromatics are water-soluble at the concentrations used in this work.

### DNA affinity purification sequencing (DAP-seq)

A DAP-seq assay was used to map SARO_RS14285 (LigR) binding sites across the genome of *N. aromaticivorans* DSM 12444. The DAP-seq experiment was carried out by the Joint Genome Institute as previously described (17, 18, 25, 30). After incubation of an individual protein with sheared *N. aromaticivorans* genomic DNA, libraries were created, and samples were sequenced on a NovaSeq 6000 S4 Flowcell (31). Briefly, the nucleotide sequences were demultiplexed, adapter-trimmed, and filtered using BBMap (https://sourceforge.net/projects/bbmap/). Filtered reads were aligned to the *N. aromaticivorans* DSM 12444 genome (GCF_000013325.1) using Bowtie 2 (v2.4.2) (32) and aligned reads were filtered using SAMtools (v1.1.1) (31). LigR DAP-seq peaks were identified using MACS3 (v3.0.0a6) (33). Data were visualized using the IGV browser (34).

### Construction of the *N. aromaticivorans* ΔLigR mutant

An ~1.2 kb fragment upstream of the SARO_RS14285 gene (*ligR*) was PCR amplified with primers 14285 UP F and 14285 UP R, and a ~1.4 kb fragment downstream of the *ligR* gene was amplified with primers 14285 DOWN F and 14285 DOWN R from *N. aromaticivorans* DSM 12444 genomic DNA. Primers used in this study are listed in Table S5, and unless stated, all protocols followed the manufacturers' specifications.

To obtain a linearized pK18*mobsacB* plasmid (35) without a multicloning site, primers pK18msB AseI ampl and pK18msB -MCS XbaI were used. The DNA fragments and the NEBuilder HiFi DNA Assembly Master Mix (New England Biolabs, Ipswich, MA, USA) were used to construct plasmid pK18.14285del. The resulting plasmid was transformed into NEB 5-alpha competent *E. coli* (New England Biolabs), cells were cultured in LB media with 50 µg/mL kanamycin, and the plasmid was purified using a Qiagen Plasmid Maxi Kit (Qiagen, Hilden, Germany). The plasmid pK18.14285del was analyzed by PCR using primers pK Insert Seq F and pK Insert Seq R, and by DNA sequencing.

Plasmid pK18.14285del was transformed into competent *E. coli* WM6026 and mobilized into *N. aromaticivorans* DSM 12444 Δ1879 by conjugation. Transconjugant *N. aromaticivorans* cells resulting from plasmid insertion in the genome were isolated on SMB media plates supplemented with 10 mM glucose and 50 µg/mL kanamycin. To select for cells that eliminated the plasmid *via* homologous recombination, transconjugant cells were cultured on SMB media containing 10 mM glucose and 10% sucrose. Deletion of the *ligR* gene was confirmed in sucrose-resistant, kanamycin-sensitive cells by PCR analysis with primers flanking *ligR*, targeted DNA sequencing across the predicted gene deletion, and whole-genome sequencing on an Illumina NextSeq 1000 sequencer (Illumina, San Diego, CA, USA) to confirm the deletion of a DNA fragment from 3,039,643 to 3,039,815 bp from *N. aromaticivorans* DSM 12444 chromosome.

### *N. aromaticivorans* growth experiments

*N. aromaticivorans* DSM 12444 and ΔLigR were cultured overnight at 200 rpm and 30°C in SMB medium supplemented with 10 mM glucose and 5 mM PCA. These cells were inoculated into SMB media with 5 mM of the indicated aromatics with and without 10 mM glucose. The cell density was measured using a Klett-Summerson photoelectric colorimeter with a red filter. For *N. aromaticivorans*, 1 Klett unit is equal to ∼8 × 10^6^ CFU/mL (10). When indicated, cells were harvested by centrifugation (5) to obtain extracellular samples for metabolite analyses.

### *N. aromaticivorans* extracellular metabolite analyses

Quantitative analysis of a set of aromatic substrates and known pathway intermediates (including SA, syringaldehyde, 3-MGA, GA, ferulic acid, vanillin, VA, 4-CA, 4-hydroxybenzaldehyde, 4-hydroxybenzoic acid, PCA, and PDC) in extracellular samples was carried out on a Shimadzu triple quadrupole liquid chromatography mass spectrometer (Nexera XR HPLC-8045 MS/MS, Shimadzu, Japan). The mobile phase was a binary gradient consisting of solvent A (water) and solvent B (0.1% formic acid in a 2:1 mixture of acetonitrile and methanol, v/v). The stationary phase was a Phenomenex Kinetex F5 column (2.6 µm pore size, 2.1 mm ID, 150 mm length, and P/N: H18-105937). All compounds were detected by multiple-reaction monitoring. Peak area and a standard curve with either commercially available compounds or PDC purified from a previously described *N. aromaticivorans* mutant (36) were used to quantify these metabolites (5, 19).

### RNA isolation and analysis of RNA-seq data

*N. aromaticivorans* parent and ΔLigR mutant strains were cultured in SMB medium supplemented with 10 mM glucose with and without 5 mM of PCA, 4-CA, VA, or SA until mid-log phase. Then, 10.875 mL of culture was used to purify RNA using hot acid phenol:chloroform extraction, as previously described (9). Remaining DNA was removed using the TURBO DNA-free Kit (Thermo Fisher Scientific, Waltham, MA, USA). Finally, the RNA was cleaned using the Qiagen RNeasy Mini Kit (Qiagen). RNA quality was analyzed with a NanoDrop spectrometer (Thermo Fisher Scientific). RNA integrity was checked in a 1% agarose gel.

RNA-seq library preparation and sequencing were performed at the Joint Genome Institute, as previously described (9, 21).

### Heterologous expression and purification of LigR

To construct plasmid pVP14285, a DNA fragment encoding the SARO_RS14285 gene was amplified by PCR with primers pJGI LysR F and pJGI LysR R from the plasmid Batch316_p053, constructed for the DAP-seq experiment by the Joint Genome Institute (18), where the *ligR* coding sequencing was codon-optimized for expression in *E. coli*. To obtain linearized pVP302K (37) without multicloning site and with 8× HisTag at the N-terminus, primers pVP ATW NoHis F and pVP ATW HisTag R were used. Methods used to construct, isolate, and verify the construction of the kanamycin-resistant plasmid pVP14285 were as described above.

Plasmid pVP14285 was transformed into competent *E. coli* Lemo21(DE3) (New England Biolabs). The resulting cells were grown at 37°C in LB media containing 50 µg/mL kanamycin and 30 µg/mL chloramphenicol until the OD_600nm_ reached 0.4. Expression of the His-tagged LigR protein was induced by adding 0.4 mM isopropyl β-d-1-thiogalactopyranoside and incubating the culture overnight at room temperature.

To purify LigR, cells were harvested by centrifugation for 15 min at 6,000 × *g* and 4°C. The cell pellet was suspended in lysis buffer (50 mM NaH_2_PO_4_·H_2_O, 100 mM NaCl, 5 mM imidazole, 10% glycerol, 5 mM TCEP, and 1% Triton X-100, pH 7.5). Cells were sonicated on ice using a QSonic sonicator set to amplitude 40 with 20 seconds on and 40 s off cycles for 15 min. After cell debris was removed by centrifugation (15 min, 13,000 × *g*, 4°C), the supernatant was applied to a Ni-NTA column (HisTrap; Cytiva, Marlborough, MA, USA) and the column was washed with a buffer (50 mM NaH_2_PO_4_·H_2_O, 200 mM NaCl, 25 mM imidazole, 5 mM TCEP, and 0.1% Triton X-100, pH 7.5). The His-tagged LigR protein was eluted using a solution of 50 mM NaH_2_PO_4_·H_2_O, 300 mM NaCl; 350 mM imidazole, 5 mM TCEP, and 0.1% Triton X-100, pH 7.5. Purity of collected fractions was analyzed by sodium dodecyl sulfate polyacrylamide gel electrophoresis (Fig. S5). The protein concentration was determined by the Bradford protein assay measuring the absorbance at 595 nm (Thermo Fisher Scientific). The purified protein was dialyzed in a Slide-A-Lyzer G3 dialysis cassette (20 KDa MWCO; Thermo Fisher Scientific) against 50 mM NaH_2_PO_4_·H_2_O, 100 mM NaCl, 10 mM imidazole, 5 mM TCEP, and 0.1% Triton X-100, pH 7.5 before storing the sample at −20°C.

### Electrophoretic mobility shift assay

DNA fragments of 200 bp containing SARO_RS14275 (*ligJ*), SARO_RS14280, SARO_RS14290 (*ligK*), SARO_RS03965 (*ligN*), or SARO_RS08360 (*ligV*) sequences were PCR-amplified from *N. aromaticivorans* DSM 12444 genomic DNA (Table S5). Except for SARO_RS14275, the DNA fragments contain the −10 and −35 sequences upstream of the known TSS. The DNA fragments were purified from 1% agarose gels using the QIAquick Gel Extraction Kit (Qiagen). DNA (20 nM) and His-tagged protein (100, 300, and 500 nM) were mixed in 50 mM Tris-HCl, 20 mM HEPES, 1 mM EDTA, 1 mM DTT, 100 mM NaCl, 10% glycerol buffer and incubated without or with purified LigR at room temperature for 30 min. The reactions were loaded on a 6% nondenaturing polyacrylamide TBE gel and electrophoresis was performed for ~1.5 h at 120 V and 4°C. The gel was stained for 1 h in the dark with a 1:10,000 dilution of SYBR green (Invitrogen, Carlsbad, CA, USA) before images were taken on a Visi-Blue transilluminator.

### DNase I footprinting

DNA fragments of ~240 bp containing upstream sequences of SARO_RS14290 (*ligK*), SARO_RS14280, and SARO_RS14275 (*ligJ*) were PCR-amplified from *N. aromaticivorans* genomic DNA (Table S5). Linearized pPK7179 plasmid was obtained by PCR using primers pPK_Lin F and pPK_Lin R (Table S5). The DNA fragments and the NEBuilder HiFi DNA Assembly Master Mix (New England Biolabs) were used to construct the plasmids pPK14290, pPK14280, and pPK14275. Isolation and sequence verification of the constructed ampicillin-resistant plasmids was performed as described above.

The above plasmids were each digested with BamHI and HindIII to release DNA fragments that were purified with phenol:chloroform (Thermo Fisher Scientific). The coding and noncoding strands of the DNA fragments were labeled with ^32^P-dGTP and ^32^P-dATP (Revvity, Waltham, MA, USA), respectively. The DNA was electrophoresed on a 5% native acrylamide gel, and regions containing ~240 bp DNA fragments were isolated and incubated overnight in 0.5 M ammonium acetate, 10 mM magnesium acetate, 1 mM EDTA pH 8.0, 0.1% SDS buffer to allow diffusion of DNA. The DNA fragments were purified with QIAquick PCR Purification Kit (Qiagen).

The DNase I assay was performed as previously described with slight modifications (38). The DNA fragments were mixed with indicated concentrations (250–5,000 nM) of His-tagged LigR. The mixtures were incubated for 30 minutes at 37°C, then DNase I (Worthington Biomedicals, Worthington, OH, USA) was added to the solution at a final concentration of 4 µg/µL, and the mixture was incubated under the same conditions for 30 s before reactions were stopped. The samples were analyzed on an 8% acrylamide gel, before radioactivity was visualized on a Typhoon phosphorimager (Cytiva).

### Homology searching in genome sequences

Homologs to SARO_RS14285 (*ligR*) and the genes surrounding it were identified using tBLASTn (v2.15.0) (39) to search for the sequences (GCF_000013325.1_RS20240116 annotation) of the surrounding genes (SARO_RS14260, SARO_RS14265, SARO_RS14270, SARO_RS14275, SARO_RS14280, SARO_RS14285, SARO_RS14290, SARO_RS14295, and SARO_RS14300) in multiple genome data sets. Default parameters were used except for -evalue 10000 and -subject_besthit. These initial settings were used to ensure that all possible homologs to *ligR* were identified before additional filtering (see below).

Two genome data sets were used to search for homologs: 4,826 representative and reference bacterial genomes as defined by NCBI on 9 January 2024, and 152 genomes used previously (9). To include high-quality matches, the tBLASTn results were filtered to include only those results with both a percent amino acid identity ≥ 30% and a query coverage value of ≥80%. NCBI taxonomy was used to summarize classes and orders of the homologs. Genomic location was determined using the left end genomic position of the match in each genome.

## SUPPLEMENTAL MATERIAL

Suplemmental Material msystems.01469-25-s0001.docx Figures S1 to S5 and Table S2.

Table S1 msystems.01469-25-s0002.xlsx Identified DAP-seq peaks for SARO_RS14285.

Table S3 msystems.01469-25-s0003.xlsx Expression levels detected in an RNA-seq for *N. aromaticivorans* parent and mutant strains in several carbon sources.

Table S4 msystems.01469-25-s0004.xlsx Homology searching of genes surrounding the LysR-type transcription factor SARO_RS14285.

Table S5 msystems.01469-25-s0005.xlsx Primers.

ASM does not own the copyrights to Supplemental Material that may be linked to, or accessed through, an article. The authors have granted ASM a non-exclusive, world-wide license to publish the Supplemental Material files. Please contact the corresponding author directly for reuse.
