# Supplementary material for: Novosphingobium aromaticivorans LigR coordinates transcription of genes involved in metabolism of multiple types of aromatics
Source: mSystems. 2025 Nov 28;10(12):e01469-25. doi: 10.1128/msystems.01469-25 (PMC12710314; doi:10.1128/msystems.01469-25)
Supplement: Suplemmental Material — Figures S1 to S5 and Table S2. [file msystems.01469-25-s0001.docx]

**Supplemental Material**

**
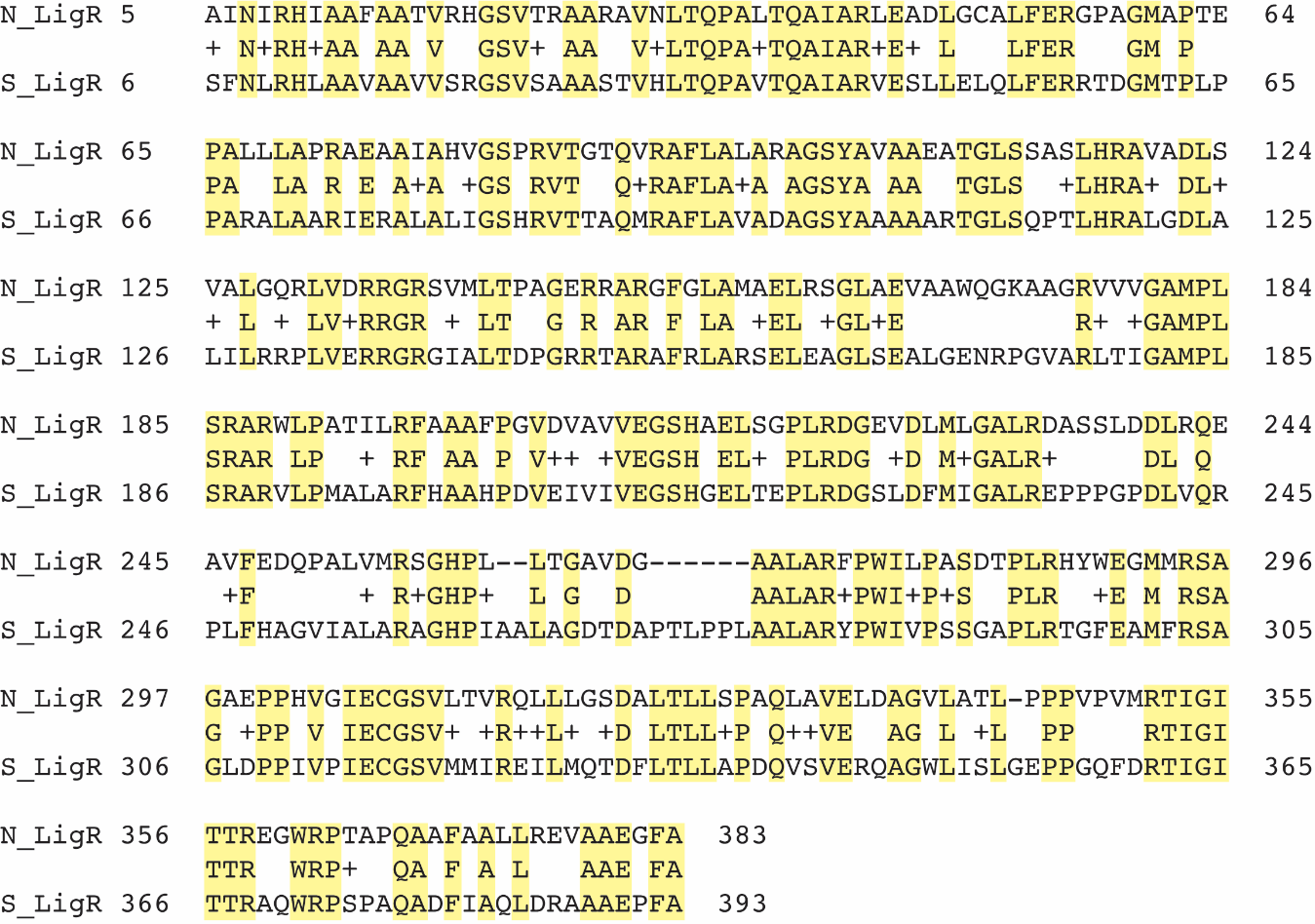
**

**Supplementary Figure 1.** Protein alignment between *N. aromaticivorans* DSM 12444 (N_LigR, SARO_RS14285, 383 AA) and *S. lignivorans* SYK-6 (S_LigR, SLG_12540, 292 AA) LigR homologs. The blastp algorithm (1) was used to perform the protein alignment of the two sequences. The percent identity and percent similarity of the two LigR homologs are 53% and 66%, respectively. Yellow boxes indicate identical amino acid residues, plus signs (+) indicate conservative substitutions, and blanks indicate no conservation between the two LigR proteins.


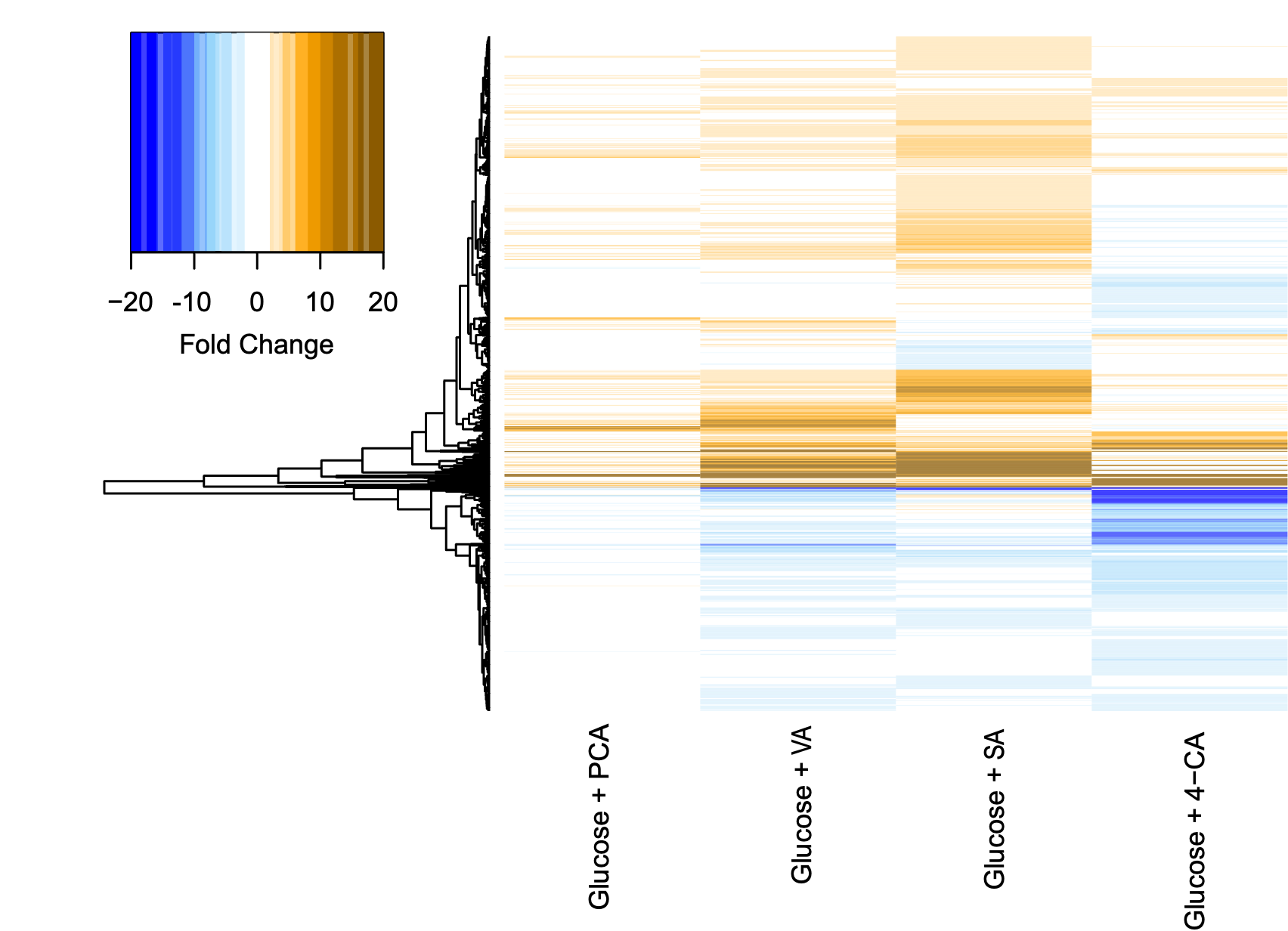


**Supplementary Figure 2.** Genome-wide changes in transcript levels when the parent strain was grown in media containing the indicated carbon sources. Cells were grown until exponential phase in the presence of 10 mM glucose without or with 5 mM PCA, 4-CA, VA or SA. The heatmap shows genes of the parent strain that increased or decreased expression when the cells were cultured in the presence of glucose and the indicated aromatics, compared to cells cultured in glucose as sole carbon source. Genes with linear change in transcript level equal to or greater than 2 and a false discovery rate (FDR) lower than 0.05 were included. Transcript abundance data used to create this figure is in Table S2.


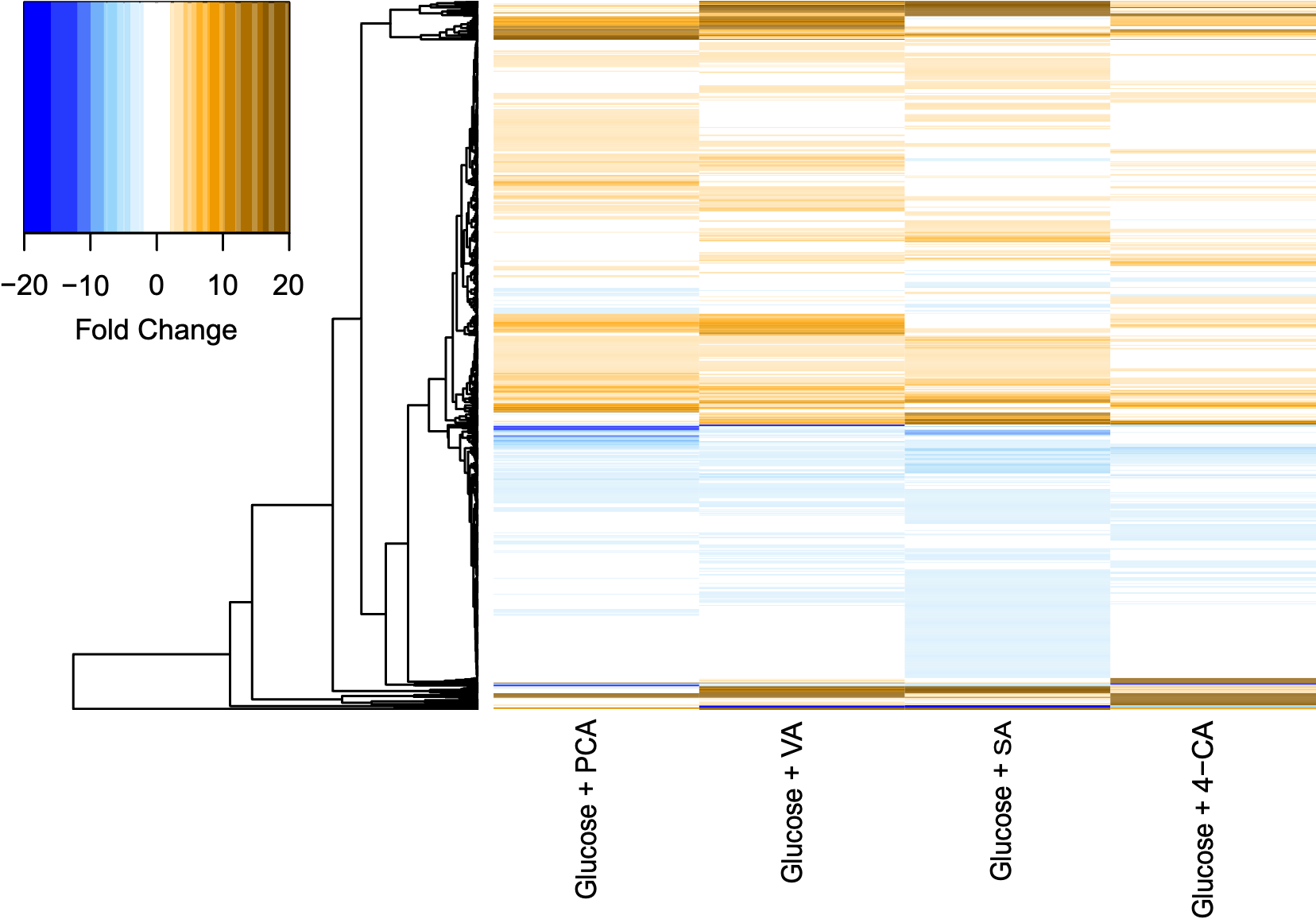
**Supplementary Figure 3.** Genome-wide changes in transcript levels when the ΔLigR mutant strain was grown in media containing the indicated carbon sources. Cells were grown until exponential phase in the presence of 10 mM glucose without or with 5 mM PCA, 4-CA, VA or SA. The heatmap shows genes of the ΔLigR strain that increased or decreased expression when the cells were cultured in presence of glucose and the indicated the aromatics, compared to cells cultured in glucose as sole carbon source. Genes with linear change in transcript level equal to or greater than 2 and a false discovery rate (FDR) lower than 0.05 were included. Transcript abundance data used to create this figure is in Table S3.


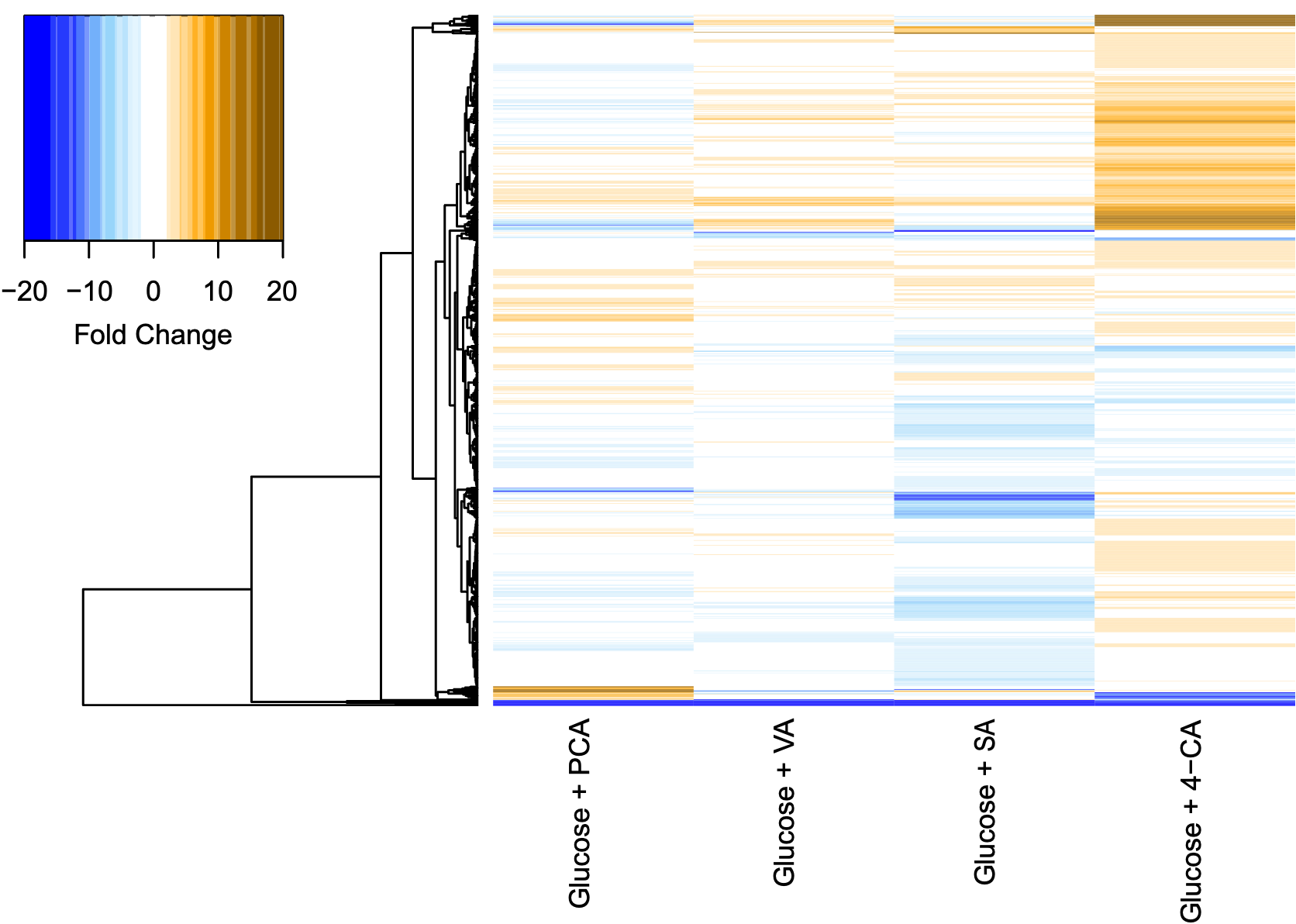


**Supplementary Figure 4.** Genome-wide changes in transcript levels when the ΔLigR mutant and parent strains were grown separately in media containing the indicated carbon sources. Cells were grown until exponential phase in the presence of 10 mM glucose without or with 5 mM PCA, 4-CA, VA or SA. The heatmap shows genes of the ΔLigR mutant strain that increased or decreased expression, compared to the parent strain. Genes with linear change in transcript level equal to or greater than 2 and a false discovery rate (FDR) lower than 0.05 were included. Transcript abundance data used to create this figure is in Table S3.


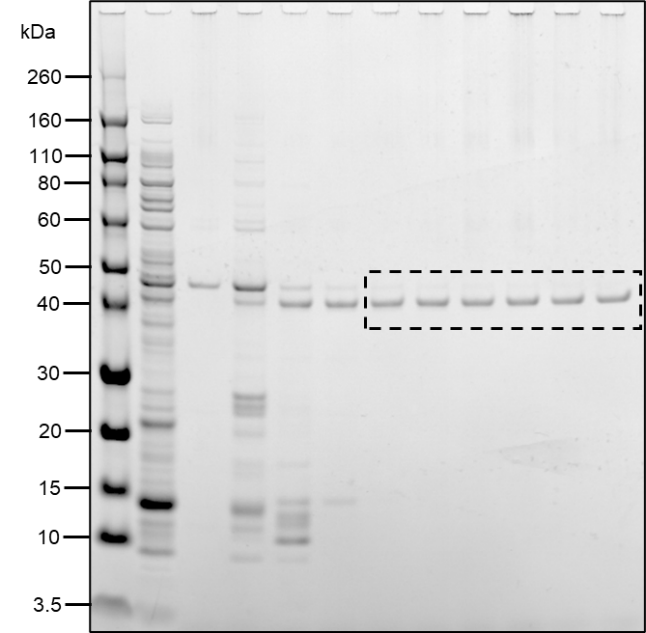
**Supplementary Figure 5.** SDS-PAGE analysis of purification of the recombinant LigR protein. A dashed square indicates the fractions collected for dialysis and DNA binding assays. (See Methods for details).

| *N. aromaticivorans*  Gene Name | *N. aromaticivorans*  Locus Tag | *S. lignivorans* SYK-6  Locus Tag | Percent Identity | Percent Similarity |
| --- | --- | --- | --- | --- |
| *ligC* | SARO_RS14260 | SLG_12490 | 87% | 92% |
| *ligB1* | SARO_RS14265 | SLG_12500 | 70% | 84% |
| *ligA1* | SARO_RS14270 | SLG_12510 | 68% | 80% |
| *ligJ* | SARO_RS14275 | SLG_12520 | 91% | 95% |
| ----- | SARO_RS14280 | SLG_12530 | 54% | 65% |
| *ligR* | SARO_RS14285 | SLG_12540 | 53% | 66% |
| *ligK* | SARO_RS14290 | SLG_12550 | 91% | 95% |
| *ligU* | SARO_RS14295 | SLG_12560 | 78% | 86% |
| *ligI* | SARO_RS14300 | SLG_12570 | 83% | 89% |
| *rpoA* | SARO_RS12725 | SLG_35380 | 88% | 92% |

**Supplemental Table 2.** Comparison of aromatic metabolism gene and regulator in *N. aromaticivorans* and homologs in *S.* *lignivoran*s SYK-6 using BLASTp (1). Percent identity and percent similarity are shown for each homolog pair.

**Reference**

1. Camacho C, Coulouris G, Avagyan V, Ma N, Papadopoulos J, Bealer K, Madden TL. 2009. BLAST+: architecture and applications. BMC Bioinformatics 10:421.
